# Interleukin-27 acts on hepatic stellate cells and induces signal transducer and activator of transcription 1-dependent responses

**DOI:** 10.1186/1478-811X-8-19

**Published:** 2010-08-19

**Authors:** Caroline Schoenherr, Ralf Weiskirchen, Serge Haan

**Affiliations:** 1 Department of Biochemistry, University Hospital RWTH-Aachen, Pauwelsstrasse 30, D-52074 Aachen, Germany; 2 Institute of Clinical Chemistry and Pathobiochemistry, University Hospital RWTH-Aachen, Pauwelsstrasse 30, D-52074 Aachen, Germany; 3 Life Sciences Research Unit, University of Luxembourg, 162A Avenue de la Faïencerie, L-1511 Luxembourg, Luxembourg

## Abstract

**Background:**

Interleukin (IL)-27 is a cytokine belonging to the IL-6/IL-12 cytokine family that is secreted by activated macrophages and dendritic cells and which strongly acts on T-cells and cells of the innate immune system. Not much is known about possible effects of IL-27 on other cell types. It signals *via *the common IL-6-type-cytokine receptor chain gp130 and the IL-27-specific chain WSX-1. We previously described that IL-27 also stimulates hepatoma cells and primary hepatocytes. The aim of this study was to investigate whether IL-27 would also act on hepatic stellate cells (HSC), the second most abundant hepatic cell type, which would demonstrate a more general role of this cytokine in the liver.

**Results:**

Using a human HSC line and primary rat HSC we investigated the signalling characteristics of IL-27 in these cells. We show that IL-27 activates signal transducer and activator of transcription (STAT) 1 and to a minor extent STAT3 in a human HSC cell line and that it leads to the induction of STAT1 target genes such as interferon response factor-1, myxovirus resistance A and STAT1 itself. Similarly we find that IL-27 also elicits STAT1-dependent responses in primary rat HSC.

**Conclusions:**

We provide the first evidence for a function of IL-27 in HSC and show that its responses resemble Interferon-γ-like functions in these cells. Our data suggests that IL-27 may play an important role in the context of liver inflammation by acting on the different liver cell types.

## Background

Liver inflammation is most often induced by viral infections, alcohol, drugs or chemical intoxication. Generally, it is associated with liver fibrosis, a wound-healing response to liver injury [1]. Among the hepatic cell types, hepatic stellate cells (HSC) are most important for this process. Activated HSC migrate and proliferate at the site of injury and perpetuate the inflammation. A key factor for the transformation of quiescent HSC into fibrogenic myofibroblasts is the cytokine transforming growth factor-β (TGF-β) [2].

Interleukin-27 (IL-27) is a type-I-cytokine belonging to the IL-6/IL-12 superfamily of cytokines [3]. It is predominantly secreted by activated macrophages and dendritic cells. As the other IL-12 family members, IL-12 and IL-23, IL-27 has profound effects on T-cells and acts on innate immune cells [4,5]. Most studies investigated the effects of IL-27 on CD4+ T-cells but not much is known about possible effects of IL-27 on other cell types. IL-27 signals *via *a receptor complex composed of the IL-27-specific receptor chain WSX-1 [3] and the common receptor subunit of IL-6-type cytokines, gp130 [6]. It is thus also a member of the IL-6-type cytokine family. We previously reported a function of IL-27 in hepatoma cells and primary hepatocytes and showed that IL-27 responses are not restricted to the classical immune cells. IL-27 was shown to exert Interferon-γ-like functions in hepatocytes/hepatoma cells and to contribute to the antiviral response in these cells [7]. The potential importance of this finding is highlighted by a recent study showing that Hepatitis B virus (HBV) enhances IL-27 expression *in vivo *and *in vitro *[8].

In the present study, we describe for the first time that IL-27 acts on hepatic stellate cells and elicits an efficient Signal transducer and activator of transcription (STAT)-1 response in these cells.

## Results

### IL-27 induces STAT1 and STAT3 phosphorylation in a human hepatic stellate cell line

**Figure 1:**
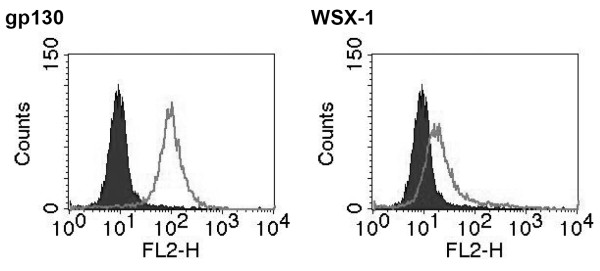
**LX-2 cells express the IL-27 receptor chains gp130 and WSX-1**. For FACS-analysis, LX-2 were incubated with a monoclonal antibody against human gp130 or human WSX-1 followed by a secondary PE-conjugated antibody. The grey histograms represent cells treated with secondary antibody only and the open histograms represent gp130 or WSX-1 surface staining.

**Figure 2:**
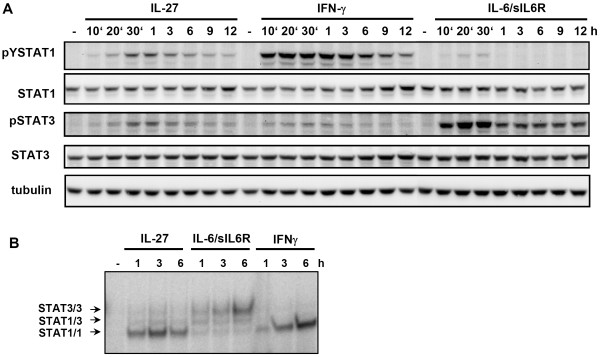
**IL-27 treatment induces STAT1 and STAT3 phosphorylation in human hepatic stellate cells**. **A: **LX-2 cells were stimulated with each 30 ng/mL IL-27, IFNγ and IL-6 together with sIL-α (500 ng/mL) for the indicated times. After lysis, cellular proteins were resolved by SDS-PAGE and tyrosine phosphorylation of STAT1 and STAT3 was detected by Western blot analysis using phospho-specific antibodies for pY701-STAT1 and pY705-STAT3. STAT protein expression was monitored by stripping and re-probing the blot with antibodies directed against total STAT1 and STAT3. Equal loading of the samples was assessed by probing the blot with an antibody against tubulin. **B: **LX-2 cells were stimulated with 30 ng/mL IL-27, IL-6 or IFNγ for the times indicated and nuclear extracts were prepared. These were analyzed by EMSA and STAT3/3, STAT3/1 and STAT1/1 dimer species were visualized by autoradiography.

Using the human LX-2 cell line, we first assessed whether these cells express both IL-27 receptor chains. This cell line retains key features of primary HSC and the gene expression profile shows strong similarities to those of primary cells (98.7%) [9]. As shown in the FACS-analysis in figure 1, we observed that both IL-27 receptor chains, gp130 and WSX-1, are expressed on these cells. Next, the cells were treated with IL-27 for up to 12 hours and tyrosine phosphorylation of STAT3 (pY705) and STAT1 (pY701) was assessed by Western blot analysis. As a control, the cells were stimulated with IFNγ or with Interleukin-6 (IL-6) together with its soluble receptor, sIL-α. IL-27 induces a sustained phosphorylation of STAT1 and STAT3 (figure 2A). As expected, IFNγ induced mainly STAT1 phosphorylation whereas IL-6 initiated a rapid and pronounced STAT3 phosphorylation. The kinetics of STAT1 and STAT3 activation by IL-27 were comparable but peaked at later time points if compared to the phosphorylation kinetics obtained after IL-6 stimulation. As previously observed IL-6 leads to a weak and transient phosphorylation of STAT1 (10, 20 and 30 min time points in figure 2A) [10]. This underlines that STAT1 phosphorylation itself is not a good indicator for the formation of active STAT1 homodimers, especially if only early time points are considered. For example, upon treatment of hepatoma cells and primary human macrophages with IL-6-type cytokines (e.g. IL-6 or OncostatinM) STAT1 phosphorylation can be observed but most of the phosphorylated STAT1 is rather trapped in STAT1/STAT3 heterodimeric complexes [10]. We thus performed electrophoretic mobility shift assays (EMSA) to examine whether phosphorylated STAT1 is forming homodimers upon treatment of LX-2 cells with IL-27 (figure 2B). As controls we used cells stimulated with IL-6/sIL-α or IFNγ. The sustained formation of STAT1/STAT1 complexes shows that IL-27 induces a persistent STAT1 activation in these cells.

### IL-27 mediates STAT1-dependent responses in human hepatic stellate cells

**Figure 3:**
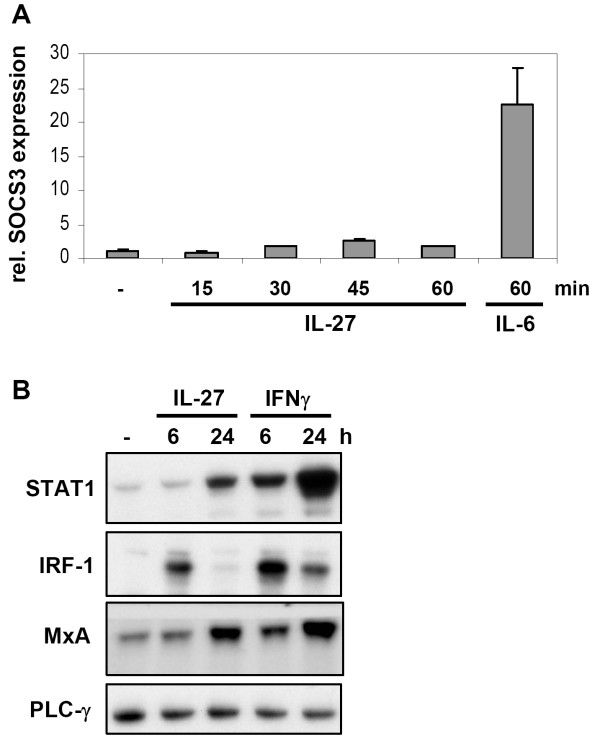
**IL-27 mediates a STAT1 response in human LX-2 cells**. **A: **Real-time PCR analysis of total cellular mRNA monitoring the expression of the STAT3-dependent gene SOCS3 in LX-2 cells after stimulation with IL-27 (30 ng/mL) for the times indicated. As a control, the cells were stimulated with IL-6/sIL6R (30 ng/mL/500 ng/mL) for 60 min. Beta-actin was used as internal reference to normalize the target transcripts. Standard deviations of a triplicate experiment are given. **B: **Western blot analysis monitoring upregulation of the STAT1-dependent STAT1, IRF-1 and MxA protein expression upon stimulation of LX-2 cells with 30 ng/mL IL-27 or IFNγ for up to 24 h. Expression levels of PLCγ are provided to compare the protein amount in the samples.

As IL-27 only led to a weak STAT3 phosphorylation and DNA-binding (figure 2), we monitored the induction of the STAT3-dependent feedback regulator suppressor of cytokine signalling (SOCS)-3 upon stimulation of LX-2 cells with IL-27 for up to 1 h. Figure 3A demonstrates that SOCS3 mRNA was hardly upregulated by IL-27 whereas the IL-6 control prominently induced SOCS3 expression. Longer treatments of the cells (up to 24 h) with IL-27 did also not further increase SOCS3 mRNA levels (data not shown).

To assess whether IL-27 induces an efficient STAT1 response, we investigated whether IL-27 would regulate STAT1-dependent gene transcription and thereby mediate interferon-like responses. We performed Western blot analyses in LX-2 cells to monitor STAT1-dependent protein expression upon treatment of these cells with IL-27 or IFNγ. Figure 3B shows that IL-27 upregulates the STAT1-dependent genes STAT1, interferon response factor-1 (IRF-1) as well as myxovirus resistance A (MxA), which is implicated in the antiviral response after IFN-treatment of cells [11]. Although protein upregulation of these genes was stronger upon treatment with IFNγ, it can be concluded that IL-27 leads to IFN-like responses in these cells.

### IL-27 induces STAT1-dependent genes in primary rat hepatic stellate cells

**Figure 4:**
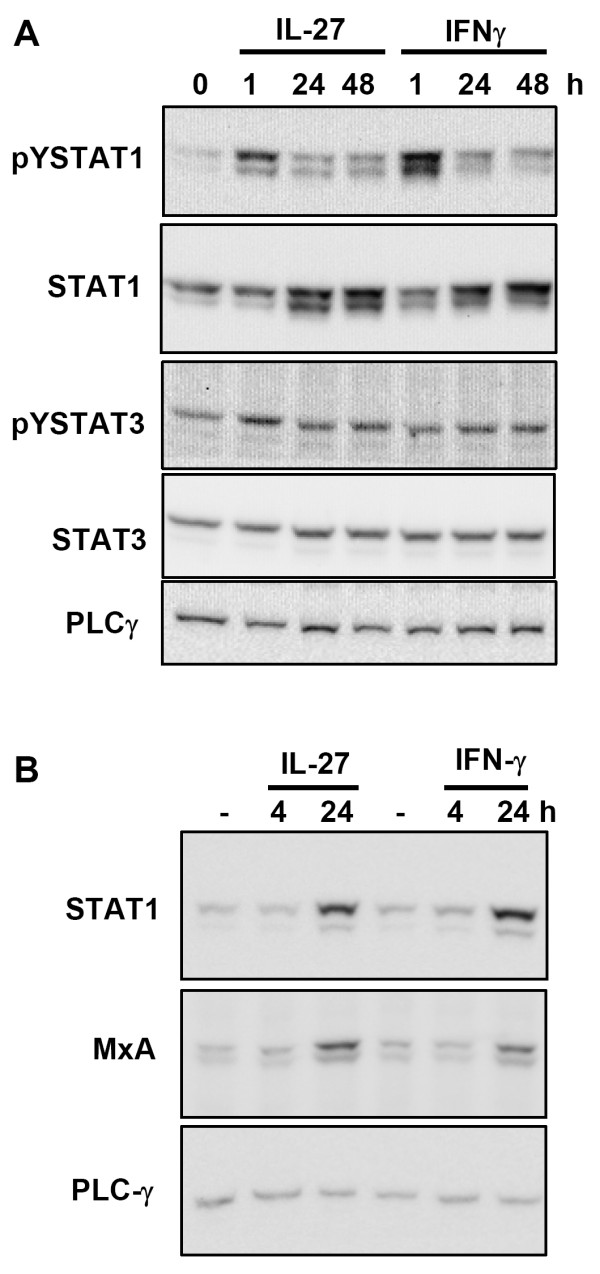
**IL-27 stimulation leads to STAT1 phosphorylation and induces STAT1-dependent protein expression in primary hepatic stellate cells**. **A: **Primary rat HSC were isolated as described in the Materials and Methods section and were treated with murine IL-27 (30 ng/mL) or with murine IFNγ (30 ng/mL) at day 5 after isolation for the times indicated. Phosphorylation of STAT1 and STAT3 was monitored by Western blot analysis as described for Figure 2. **B: **Western blot analysis monitoring upregulation of STAT1 and MxA protein expression upon stimulation of primary rat HSC with 30 ng/mL IL-27 or IFNγ for up to 24 h. Expression levels of PLCγ are provided to compare protein loading in the individual samples.

In order to test whether IL-27 also acts on HSC in primary culture we isolated rat HSC and stimulated them with IL-27 or IFNγ for different times. As illustrated in figure 4A, treatment of these cells with IL-27 or IFNγ leads to a sustained phosphorylation of STAT1. In comparison, IL-27 only leads to a weak increase in STAT3-phosphorylation after 1 hour. Furthermore, as already observed in the HSC line (i.e. LX-2), IL-27 upregulates the STAT1-dependent proteins STAT1 and MxA in primary HSC and thereby induces a response resembling that of IFNγ (figure 4B).

### IL-27 stimulation does not affect the expression of Smad2 and Smad3 or the activation of a Smad-dependent reporter construct

**Figure 5:**
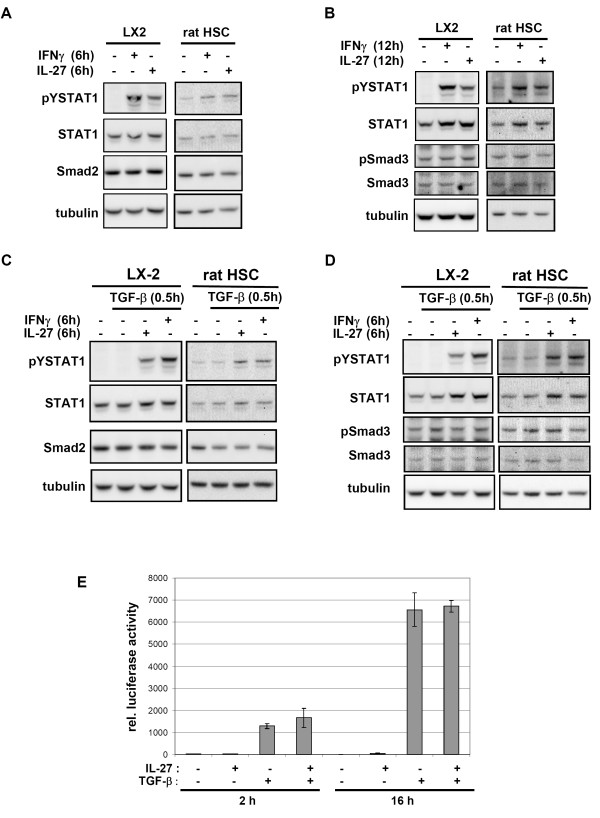
**IL-27 stimulation does not affect TGF**-**β-mediated activation of Smad proteins in LX2 cells and rat HSC**. **A, B: **LX-2 cells or rat HSC were pretreated with IL-27 (30 ng/mL) or IFNγ (30 ng/mL) for 6 h (**A**) or 12 h (**B**). Expression of Smad2 (**A**) or phosphorylation and expression of Smad3 (**B**) was monitored by Western blot analysis. Staining for tubulin, pY-STAT1 and STAT1 are provided as controls. **C, D: **LX-2 cells or rat HSC were pretreated with IL-27 (30 ng/mL) or IFNγ (30 ng/mL) for 6 h (**C**) or 12 h (**D**) and were subsequently stimulated with TGF-β (1 ng/mL) for 0.5 h. Expression of Smad2 (**C**) or phosphorylation and expression of Smad3 (**D**) was monitored by Western blot analysis. Staining for tubulin, pY-STAT1 and STAT1 are provided as controls. **E: **Induction of a Smad2/Smad3/Smad4-dependent CAGA reporter construct after treatment of LX-2 cells with IL-27 (30 ng/mL), TGF-β (1 ng/mL) or both for 2 h or 16 h. Standard deviations for a triplicate experiment are given as error bars.

It was recently reported that the TGF-α/Smad3 pathway is accelerated in STAT1-deficient mice [12]. In addition, Weng et al. reported that prestimulation of rat HSC with IFNγ for 6 h or 12 h impairs the intrinsic phosphorylation of Smad2 and Smad3 that occurs by autocrine TGF-β stimulation [13]. Furthermore, they found Smad2 and Smad3 expression to be reduced to a comparable degree in these conditions. The effects on Smad2 were most prominent after a pretreatment of 6 h whereas Smad3 was most affected after a 12 h prestimulation [13]. We performed similar experiments to investigate whether stimulation of LX-2 cells or rat HSC with either IFNγ or IL-27 would affect Smad2 and Smad3 expression. As shown in figure 5A and 5B, we did not observe reduced expression of either Smad2 or Smad3 although STAT1 was clearly activated. Similarly we did not detect a clear reduction of the intrinsic Smad3 phosphorylation (figure 5B). We repeated the experiment and additionally treated the cells with TGF-β following pretreatment with IL-27 or IFNγ (figure 5C and 5D). Similarly, we did not detect a reduction of Smad2 or Smad3 expression levels. TGF-β stimulation slightly increased Smad3 phosphorylation (figure 5D) but prestimulation with either IL-27 or IFNγ did not impair this activation. A reduction in Smad3 phosphorylation was always paralleled by a reduction in the loading control (tubulin detection). To further substantiate this finding, we performed a reporter gene analysis using a CAGA-reporter construct that binds the Smad2/Smad3/Smad4 complex after phosphorylation of Smad2 and Smad3. The cells were stimulated with TGF-β1 or IL-27 alone or in combination with both cytokines. We observed an increase in luciferase activity upon treatment of the cells with TGF-β1 for 2 or 16 hrs but this induction was not inhibited by IL-27 co-stimulation (figure 5E). Taken together, we could not confirm inhibitory effects of IFNγ prestimulation on Smad2 and Smad3 expression or Smad3 phosphorylation. Similarly, pretreatment with IL-27 did not affect these parameters.

## Discussion

Interleukin-27 was previously described to be involved in inflammatory processes within the gastrointestinal tract. It was shown to play a key role in the context of Con-A-induced hepatitis [14], Crohn's Disease [15] as well as in the initiation and progression of colon carcinoma [16,17]. Furthermore, IL-27 was recently reported to possess strong antitumor activity in a murine model of hepatocellular carcinoma [18]. These studies highlight the importance of IL-27 expression in the gastrointestinal tract and the liver. However, the reported functions of IL-27 were all restricted to infiltrating immune cells such as T-cells and natural killer cells. Recently, we reported a first function for IL-27 on a hepatic cell type, namely hepatocytes [7]. Here we investigated whether the second most abundant liver cell type (i.e. HSC) also responds to IL-27. We show that IL-27 induces a STAT1 response in these cells and upregulates proteins involved in antiviral responses.

The members of the IL-6 type family of cytokines play important roles in the liver as they contribute to the acute-phase response of the liver as well as liver regeneration [19-21]. These functions of IL-6-type cytokines are largely dependent on the activation of the transcription factor STAT3 and the formation of activated STAT3/STAT3 dimers. The activation of STAT3 is mediated *via *the IL-6-type cytokine receptor chains gp130, oncostatinM receptor and leukemia inhibitory factor receptor [6]. Although these receptors can also recruit STAT1 and lead to its phosphorylation, IL-6-type cytokines such as IL-6 and OSM fail to induce an efficient STAT1 response in cell types such as hepatocytes and macrophages [10,22,23]. It was reported that the majority of the phosphorylated STAT1 is trapped in STAT1/STAT3 heterodimers [10]. This provides an explanation for the fact that IL-6 and OSM induce an interferon-like response in STAT3 knock-out cells [22,23]. In this case, the lack of STAT3 prevents the formation of heterodimers and will thereby lead to the formation of STAT1 homodimers.

IL-27 signals *via *a signalling complex containing the STAT3 activating receptor chain gp130 and a STAT1 activating receptor chain WSX-1 [24]. Here we report that IL-27 mediates an efficient STAT1 response in HSC (figures 3B and 4). Furthermore, we only found a weak induction of a STAT3-dependent gene, SOCS3 (figure 3A). In classical immune cells such as CD4+ T cells and macrophages, the importance of STAT1 for the various biological activities of IL-27 was shown [25-27]. However, it was also reported that IL-27 activates both STAT1 and STAT3 in early activated T-cells whereas it displays a preferential activation of STAT3 in fully activated CD4+ T cells [28]. This suggests that specific IL-27 responses may be the result of differently regulated STAT1 and STAT3 responses and that the extent of STAT3 activation (*via *gp130) and STAT1 activation (*via *WSX-1) may depend on the cell type and/or on the activation status of the cell. Such a preference for STAT1 or STAT3 responses could solely be due to different STAT expression levels (and thus a different distribution of STAT-dimer species), differences in Janus kinase activation by specific receptor chains or it could for example depend on the involvement of specific regulatory proteins. As we consistently observe a much lower STAT3 activation by IL-27 in hepatocytes and hepatic stellate cells compared to IL-6 one can also speculate that the STAT3 activation after IL-27 stimulation does not reach a necessary threshold for efficient induction of STAT3-dependend genes such as SOCS3. Also, the delayed kinetics of STAT3 phosphorylation if compared to IL-6 may contribute this effect (figure 2A). It must be noted however that in HSC SOCS3 upregulation was also not apparent at later time points following IL-27 stimulation (data not shown). Our present data fits to our previous observation that IL-27 does not regulate the STAT3-dependent acute-phase proteins γ-fibrinogen and hepcidin in hepatocytes and hepatoma cells [7]. The data suggest that the STAT1 response is the most important feature in IL-27 stimulated liver cells. The sustained and efficient STAT1 response upon stimulation of cells with IL-27 is mediated via the WSX-1 chain of the receptor complex [24]. WSX-1 contains a single conserved tyrosine motif (Y_609_EKHF) which resembles the STAT1-recruiting motif of the Interferon-gamma receptor 1 (Y_440_DKPH)[29]. Interestingly, this motif clearly differs from the tyrosine motifs of gp130 that are reported to recruit STAT1 (Y_905_LPQ, Y_915_MPQ) [30]. As these motifs (Y_905 _and Y_915_) are also the motifs that bind STAT3 with the highest affinity [31] it is conceivable that the competition between STAT1 and STAT3 at these motifs is responsible for the inefficient STAT1 activation via the gp130 chain and/or the preferred formation of STAT1/STAT3 homodimers [10].

It was reported that IL-27 can have antiviral activities in PBMCs, CD4+ T cells and macrophages and that it can inhibit HIV-1 replication [27,32]. It can induce IFN-inducible antiviral genes such as myxovirus protein 1, 2'-5'-oligoadenylate synthetase 2 and RNA-dependent protein kinase in macrophages, suggesting that IL-27 inhibits HIV-replication by eliciting an interferon-like response [27]. Similarly, IL-27 has antiviral activity in hepatoma cells and can induce the expression of IRF-1, guanylate binding protein 2 and MxA proteins that are involved in the antiviral response [7]. Importantly, it was recently reported that patients suffering from a hepatitis B infection have elevated IL-27 serum levels. Furthermore, IL-27 levels were also enhanced in patients with liver cirrhosis or hepatocellular carcinoma [8]. Here we show that IL-27 upregulates the STAT1-dependent genes STAT1, IRF-1 and MxA in HSC and that it initiates an IFN-like response in these cells. Our data indicate that IL-27 has wide-spread activities in the liver as it induces genes involved in host resistance to pathogens in the two most prominent hepatic cell types. IL-27 may thus be a suitable candidate for studies on combination therapies against hepatitis C, especially in light of the fact that novel IFN-based products are currently being developed [33].

The potential importance of our observations for liver inflammation is further supported by a recent report which provides evidence that liver dendritic cells produce high amounts of IL-27 instead of IL-12 upon endotoxin exposure [34]. In this experimental setting, the secreted IL-27 would of course affect responding liver cells such as hepatocytes and HSC.

STAT1 is discussed to be a negative regulator in liver fibrosis and its activation was reported to inhibit signalling by TGF-β, one of the major players involved in liver fibrosis [12,13]. This cytokine is a key mediator of fibrogenesis because of its ability to induce the transition of HSC to contractile myofibroblasts and to initiate the production of extracellular matrix proteins [2]. We thus investigated whether IL-27 stimulation of HSC would inhibit TGF-β/Smad2/Smad3 signalling. Such an effect would be of great interest because many antifibrotic drugs aim at the inhibition of HSC activation and proliferation [1]. As reported by others [13], we investigated whether prestimulation with IFNγ would reduce Smad2 and Smad3 expression. Additionally, we also studied the effects of IL-27 prestimulation on LX-2 cells and rat HSC. However, an inhibitory effect of either pretreatment on TGF-β-mediated Smad2 and Smad3 expression was not apparent in our experiments (figure 5A, B, C and 5D). Nevertheless, it must be noted that the STAT1-dependent effects on TGF-β mediated fibrotic responses may be multifaceted and occur at various levels. For example, Ghosh et al. proposed that IFNγ activated STAT1 inhibits Smad3 activity by competing for the interaction with the transcriptional coactivators CBP and p300 [35]. In addition, IFNγ has also been shown to induce the Smad7, a negative regulator of TGF-β signalling [13]. Further detailed studies will be necessary to clarify whether IL-27 can counteract the profibrotic effects of TGF-β.

## Conclusion

Taken together, we show for the first time that IL-27 acts on hepatic stellate cells and that it regulates STAT1-dependent genes in this hepatic cell type. Together with our observation that IL-27 also acts on hepatocytes, a general role for this cytokine in the liver becomes obvious. We show that the liver cells can contribute to antiviral and inflammatory responses via an IL-27-induced STAT1 activation. Further studies will have to deal with the complex interplay between the various IL-27 responsive inflammatory cells (e.g. dendritic cells, T-cells) and the liver cells in order to dissect the precise role of IL-27 in liver inflammation.

## Methods

### Cell culture

Human HSC line LX-2 (kind gift of Scott L. Friedman) was maintained in DMEM/Nut.MixF-12 medium with Glutamax supplemented with 2% FBS, 100 mg/L streptomycin, and 60 mg/L penicillin. All human and murine cytokines used in this study were obtained from R&D Systems. For cross-talk experiments, the cells were starved for 12 h and then prestimulated with IFNγ or IL-27 for 6 h or 12 h. Subsequently, the cells were stimulated with TGF-β for 30 min.

### Isolation and cultivation of rat hepatic stellate cells

Rat hepatic stellate cells were isolated following a protocol that is based on enzymatic collagenase and pronase digestion of the liver followed by centrifugation of the crude cell suspension through a Nycodenz gradient. Details about the purification, seeding, culturing and estimation of purity were described previously [36]. This two-step protocol yields routinely in approximately 3-5 × 10^7 ^cells per rat. Experiments were performed at day 5 after isolation. All experiments were approved by the *Landesamt für Natur, Umwelt und Verbraucherschutz NRW *(LANUV), Recklinghausen (AZ 8.87-50.10.45.08.206).

### Reporter gene analysis

Reporter gene analysis was performed as previously described [7]. The pGL3-(CAGA)_12_-luc construct (kind gift of P. ten Dijke) contains a repeat of 12 CAGA elements that mediate Smad-binding [37]. TGF-β1 stimulation experiments in LX-2 and rat HSC cells were done with a concentration of 1 ng/ml recombinant human TGF-β1 for indicated time intervals.

### Cell lysis, Western blot analysis and antibodies

Cells lysis and Western blot analysis were performed as described previously [38]. The following antibodies were used: anti-STAT3, anti-STAT1 (#610190, #610116, Transduction Laboratories), anti-pYSTAT3, anti-pYSTAT1, anti-PLCγ, anti-phospho-Smad3(Ser423/425), anti-Smad3, anti-Smad2 (#9131, #9171, #2822, #9520, #951, #3103, Cell Signalling), anti-IRF-1, anti-MxA (sc-497, sc-50509, Santa Cruz Biotechnologies), and anti-tubulin (Sigma). HRP-conjugated secondary antibodies were purchased from DAKO. Signals were detected using the ECL system (Amersham Pharmacia Biotech).

### Detergent-free preparation of nuclear extracts and Electrophoretic Mobility Shift Assay (EMSA)

The preparation of nuclear extracts and the EMSA were essentially performed as previously described [10]. Protein concentrations of nuclear extracts were measured using a NanoDrop spectrophotometer (PEQLAB). The DNA-bound STAT complexes were visualized using a Typhoon phosphorimager (Amersham Pharmacia).

### Real time PCR analysis

Total mRNA was isolated with the RNeasy Kit (Qiagen) according to the manufacturer's instructions. The purified RNA was reverse transcribed with the Omniscript RT Kit (Qiagen) using random hexamer primers. Real-time PCR was carried out on an ABI PRISM 7000 Sequence Detection System (Applied Biosystems) using the following primers: β-actin: sense 5'-CCC TGA GGC ACT CTT CCA G-3', antisense 5'-TGC CAC AGG ACT CCA TGC CC-3'; SOCS3: sense 5'-CAC CTG GAC TCC TAT GAG AAA GTC A-3', antisense 5'-GGG GCA TCG TAC TGG TCC AGG AA-3'. The relative differences between the cytokine-stimulated and control samples were calculated with the mathematical Pfaffl model established for relative quantification in real-time RT-PCR [39].

### Flow cytometry

5 × 10^5 ^to 1 × 10^6 ^cells were harvested in cold PBS supplemented with 10 mM EDTA and 0.1% sodium azide. They were then resuspended in 100 μl PBS/azide (PBS, 5% FBS, 0.1% sodium azide) and incubated with 1 μg/ml of monoclonal anti-human TCCR/WSX-1 (R&D Systems) for 30 min at 4°C. Cells were then washed with cold PBS azide. To visualize the bound antibodies, the cells were subsequently incubated in darkness with a 1/100 dilution of a R-phycoerythrin-conjugated anti-mouse IgG-Fab (Dianova, Hamburg) for 30 min at 4°C. Cells were again washed with cold PBS-azide and then 10^4 ^cells per sample were analyzed by flow cytometry using a FACSCalibur flow cytometer (Beckton Dickinson) equipped with a 488 nm argon laser.

## Competing interests

The authors declare that they have no competing interests.

## Authors' contributions

CS performed experiments in LX2 cells and primary rat hepatocytes and helped to draft the manuscript. RW participated in the design of the study, isolated and cultivated rat hepatic stellate cells and helped to draft the manuscript. SH conceived the study, performed experiments in primary rat HSC and LX2 calls and drafted the manuscript. All authors read and approved the manuscript.
